# ASO Author Reflections: PIPAC Oxaliplatin in Refractory Colorectal or Appendiceal Carcinomatosis: Encouraging Results from a Phase 1 Clinical Trial

**DOI:** 10.1245/s10434-023-14067-1

**Published:** 2023-08-05

**Authors:** Kevin M. Sullivan, Thanh H. Dellinger, Mustafa Raoof

**Affiliations:** City of Hope National Medical Center, Department of Surgery, Duarte, CA USA

## Past

Most patients with peritoneal metastases from colorectal cancer (CRC) or high-grade appendiceal cancer are not candidates for cytoreductive surgery. Systemic therapy has been the standard of care; however, the efficacy is limited. For instance, first-line systemic chemotherapy alone is less effective for peritoneal carcinomatosis from CRC compared with non-peritoneal metastatic sites, with median overall survival (OS) of 12.7 months and progression-free survival (PFS) of 5.8 months.^1^ Most patients will succumb to the regional complications of disease progression, and therefore there is a strong rationale for regional therapy. Pressurized intraperitoneal aerosolized chemotherapy (PIPAC) has emerged as a novel way to deliver intraperitoneal chemotherapy with several prospective studies from the USA and Europe demonstrating safety and feasibility of PIPAC with different drug regimens. For instance, the largest prospective trial of oxaliplatin use in PIPAC, PIPAC-OPC2,^2^ demonstrated low rates of surgical complications and CTCAE grade ≥ 3 adverse events. This study and several others included multiple different gastrointestinal malignancies, including small bowel, gastric, or hepatobiliary cancers in addition to CRC and appendiceal cancers, and prior chemotherapy exposure was not standardized.

## Present

The current study^3^ represents the first trial of PIPAC in 12 patients in the USA and demonstrates the safety and feasibility of oxaliplatin PIPAC in heavily pre-treated patients with CRC or appendiceal cancer with unresectable peritoneal carcinomatosis. Compared with prior studies, patients in this study had a more homogenous prior chemotherapy exposure with a median of two prior lines of chemotherapy. The trial included patients with peritoneal metastases from CRC and non-low grade appendiceal cancers, which are treated very similarly with respect to systemic therapy. This homogeneity in inclusion criteria allows for an improvement in interpretation of efficacy. While not powered to demonstrate efficacy of oxaliplatin PIPAC, the study did show promising results with respect to treatment effectiveness, including median OS of 12.0 months and median PFS of 2.9 months, with two patients who went on to receive cytoreduction. A secondary analysis showed that radiographic response was associated with survival but assessment of response by laparoscopy or histologic grading were not, a finding that may have implications in selecting endpoints for future prospective studies.

## Future

The current and prior studies have established safety and demonstrated an encouraging efficacy signal. These studies have set the stage for a prospective randomized trial of PIPAC oxaliplatin versus best available systemic therapy for patients with chemotherapy refractory colorectal or appendiceal cancer. While the preliminary efficacy signals are encouraging, the authors strongly believe that a randomized trial is necessary before widespread adoption of PIPAC.
